# Fractal-structured multifocal intraocular lens

**DOI:** 10.1371/journal.pone.0200197

**Published:** 2018-07-09

**Authors:** Laura Remón, Salvador García-Delpech, Patricia Udaondo, Vicente Ferrando, Juan A. Monsoriu, Walter D. Furlan

**Affiliations:** 1 Departamento de Física Aplicada, Universidad de Zaragoza, Zaragoza, Spain; 2 Ophthalmology Department, Hospital Universitario La Fe, Valencia, Spain; 3 Centro de Tecnologías Físicas, Universitat Politècnica de València, Valencia, Spain; 4 Departamento de Óptica y Optometría y Ciencias de la Visión, Universitat de València, Burjassot, Spain; Universidade do Minho, PORTUGAL

## Abstract

In this work, we present a new concept of IOL design inspired by the demonstrated properties of reduced chromatic aberration and extended depth of focus of Fractal zone plates. A detailed description of a proof of concept IOL is provided. The result was numerically characterized, and fabricated by lathe turning. The prototype was tested in vitro using dedicated optical system and software. The theoretical Point Spread Function along the optical axis, computed for several wavelengths, showed that for each wavelength, the IOL produces two main foci surrounded by numerous secondary foci that partially overlap each other for different wavelengths. The result is that both, the near focus and the far focus, have an extended depth of focus under polychromatic illumination. This theoretical prediction was confirmed experimentally by means of the Through-Focus Modulation Transfer Function, measured for different wavelengths.

## Introduction

With millions of procedures carried out each year, cataract surgery is one of the most common operations nowadays, with an increasing rate of growth worldwide. Cataracts frequently start to develop in people as they get older, producing a loss of vision that can only be corrected by surgery. In cataract surgery, the crystalline lens that has become cloudy, is removed and replaced with an intraocular lens (IOL). Many of the IOLs that are currently in the market are bifocals designed to provide good distance and near vision. Depending on the lens design, several addition powers, distribution of energy between the foci, and depth of focus are available with different models [1]. However, the main shortcoming of current bifocals is their low performance at intermediate distances [2,3]. Therefore, due to the patient’s demand, nowadays there is a trend to design new IOLs that provide also good intermediate vision, which is important for performing several daily tasks (such as, viewing the dashboard in a car, cooking, using computers and smartphones, etc). This tendency was initiated a few years ago with the introduction of the low-addition bifocal IOLs [3,4], intended to match the lens addition with the patient’s intermediate focus. Diffractive trifocal IOLs were introduced later with the aim to offer simultaneously two different additions, one (+3.50D), for near vision and the other (+1.75D) for intermediate vision [5].

Following the above mentioned trend, more recently both, refractive, and diffractive, extended depth of focus (EDOF) designs have been developed with the intention to provide a “continuous” range of vision, from far to intermediate-near vision. In the first group, the refractive zones in the lens, having different powers, can be either: rotationally symmetric in different annuli, like the M-flex multifocal IOL (Rayner, Hove, United Kingdom), or angularly segmented, like the Lentis M-Plus (Oculentis GmbH, Berlin, Germany), and the SBL-3 IOL (Lenstec, St. Petersburg, USA). In the second group, two new diffractive multifocal IOLs were designed; the Mini WELL Ready (SIFI MedTech, Catania, Italy) and the TECNIS Symfony ZXR00 (Abbott Laboratories, Illinois, USA). These last two models are based on different optical principles. The Mini WELL Ready presents different amounts of spherical aberration in two concentric zones in the central part of the lens [6]. The TECNIS Symfony ZXR00 is based on the combined correction of the spherical and longitudinal chromatic aberrations of the eye [7]. In truth, as recently reported by Millán and Vega [8], the in vitro EDOF performance of this IOL is highly wavelength-dependent. On the other hand, a previous study [9] showed that, even for monochromatic light (545 nm), the EDOF of both diffractive designs is also pupil-dependent.

In this work we present a conceptually new multifocal IOL design intended to provide good vision at multiple distances. A proof of concept of a multifocal IOL was constructed following a hybrid diffractive-refractive design [10] that provides EDOF and low chromatic aberration simultaneously. These properties are inherited from Fractal zone plates (FZPs) and devil’s lenses [11–15] which are diffractive lenses that have multiple foci with unique self-replicating fractal structure around a main focus. Under white light illumination, different wavelengths come to focus at different distances, but with certain degree of overlapping that results in an EDOF with reduced chromatic aberration. The fractal design can also be used to modify both the number and the relative intensities of the foci. FZPs have been successfully employed in several areas, ranging from spectral-domain optical coherence tomography [16] to terahertz technology [17]. Here we expand the range of applications of fractal lenses by presenting a novel design of multifocal IOL developed using the fractal triadic Cantor set. This set is used to modify the pure spherical profile of a monofocal IOL so that the resulting refractive-diffractive hybrid design has two main powers, intended for distance and near vision, with EDOF for intermediate vision. Thus, we called it: Fractal Intraocular Lens (FIOL). The FIOL proof of concept was numerically evaluated, and tested in vitro on an optical bench.

## Intraocular lens design and construction

**Fig 1:**
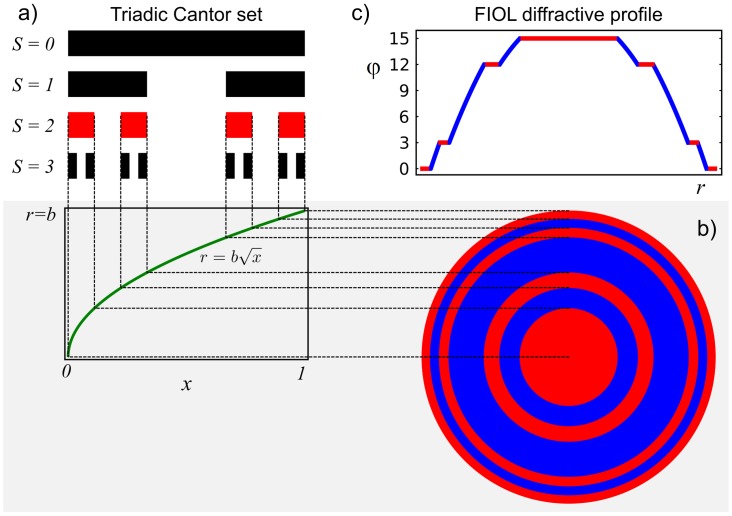
FIOL design. a) Top left: Triadic Cantor set developed up to three steps, *S* = 3; b) FIOL fractal zones distribution for *S =* 2, obtained through the coordinate transformation *r = b√(x)* c) FIOL diffractive profile obtained with *K* = 3 (see the main text for details).

FZPs are characterized by the distribution of the annular diffractive zones they have, which, in spite of being periodic along the square root of the radial coordinate, like a Fresnel zone plate is, it follows the sequence of a given fractal Cantor set. In previous works we have demonstrated that FZPs can be constructed following any class of Cantor Functions, including polyadic Cantor sets [18] and functions with variable lacunarity [19]. As a proof of concept, our first FIOL design is based on the simplest Cantor set shown in Fig 1a, which is called triadic Cantor set. The first step in the construction procedure of this set, consists in defining a straight-line segment of unit length, called *initiator* (stage *S* = 0). Next, at stage *S* = 1, the *generator* of the set is created by dividing the segment into 3 equal sub segments of length *x* = 1/3 and removing the central one. This procedure is repeated for the subsequent stages, *S* = 2, 3…, on each sub segment. Then, a change of variables *r = b√(x)* is performed to define the extension of the concentric zones in the FIOL, up to a given lens radius *b* (see Fig 1b). In this way, our design alternates annular zones that follows a fractal distribution along the square of the radial coordinate. Note that the total number of sub-segments in each stage of the Cantor set is N = *3*^*S*^; and that each one of them, with an extension *x* = 1/3^S^, has a corresponding Fresnel zone in the FIOL fractal zone distribution. The next step in the FIOL design process is to define the phase profile of these zones in such a way that the first diffraction order of this structure will produce the near FIOL power. One solution is to employ a conventional kinoform profile in which the facets of the lens produce a 2π phase shift for the design wavelength λ. These lenses, known as devil’s lenses, have a focal distance that depends on the number of the above mentioned Fresnel zones, through the *S* parameter as *f* = *b*^*2*^/2 λ_0_*3*^*S*^ [12]. In this way, the FIOL addition (*Ad*), i.e.; the difference between the near and far powers results:

$$Ad\;=\;\frac{2\;\lambda_{0}3^{S}\;}{b^{2}}$$ (1)

However, for our purposes is convenient to introduce one more degree of freedom in the FIOL design, to cover a wide range of *Ad*s with the same fractal structure. By using the concept of harmonic diffractive lens [20], this is possible if the phase difference introduced in each Fresnel zone is φ = 2π*K*, being *K* a positive integer number. Additionally, to facilitate the lens construction, the above mentioned phase differences can be “staked” sequentially from the periphery to the center avoiding a saw tooth (kinoform) profile. In this way, in each Fresnel zone, the increment of height corresponding to the desired *Ad* is *Δh* = *K λ*_0_/ (*n*–*n’*), where *n* and *n’* are the refractive index of the lens material and the surrounding FIOL media (aqueous humor) respectively. Therefore, the FIOL *Ad* can be expressed alternatively as:

$$Ad\;=\;\frac{2\;3^{S}\Delta h(n-n’)\;}{b^{2}}$$ (2)

As reported in Ref [20], lenses constructed in this way have hybrid properties of both refractive and diffractive lenses.

Returning to Fig 1, if we choose S = 2 in the Fractal structure, and considering a “center far” FIOL design, the *Ad* phase profile is incremented in the “blue” rings in Fig 1b). For *K* = 3 the final result is shown in Fig 1c).

**Fig 2:**
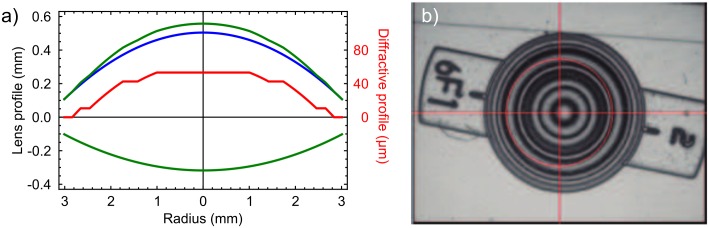
FIOL proof of concept. a) Theoretical profiles of the anterior and posterior FIOL surfaces (green line). The red line is the diffractive profile of the FIOL, designed with *S* = 2 and *K* = 3 (magnified X5 in the vertical direction in order to show the relative heights of the diffractive steps); this profile was superimposed to a pure spherical profile of a monofocal IOL radius *r* = 12.42 mm (blue line). b) Interferometric image of the constructed lens.

A FIOL prototype was designed to be constructed in Polymethyl methacrylate (PMMA) (refractive index *n* = 1.493 at the design wavelength λ_0_ = 555x10^-9^m); with dioptric power 19.5 D. The radii of curvature for the front and back surfaces were 12.42x10^-3^m, and 22.89x10^-3^m respectively. The proof of concept FIOL was conceived with the fractal profile in the anterior surface of the lens providing an *Ad* = +3.5 D. This value was obtained with: *S* = 2, *K* = 3, and *b* = 2.92x10^-3^m using Eq (1). See Fig 2a.

The multifocal FIOL was manufactured by a lathe-milling process (Optoform40, Sterling Ultra Precision, Largo FL, USA), similar to that for standard monofocal IOLs, but without the polishing step. Differences between the theoretical design and the constructed FIOL profiles were lower than 0.1 mm as measured with an optical non-contact profilometer (PLμ 2300, SENSOFAR, Terrassa, Spain). An interferometric image (PMTF, Lambda-X, Nivelles, Belgium) of the manufactured FIOL is shown in Fig 2b. The haptic for the prototype was chosen as shown in the figure, simply to facilitate the lens handling during its assessment (the design of the lens haptic has no influence on its optical properties and it was beyond the scope of this work).

## Numerical validation of the FIOL design

For the theoretical characterization of the lens, wavefront propagation and Fourier analysis were performed numerically using Fresnel diffraction theory. In the simulations, it is assumed that the lens is immersed in aqueous humor (refractive index: *n’* = 1.336). To assess its focusing properties, the Point Spread Function (PSF) provided by the FIOL, was computed at different axial positions for different pupil diameters and wavelengths.

**Fig 3:**
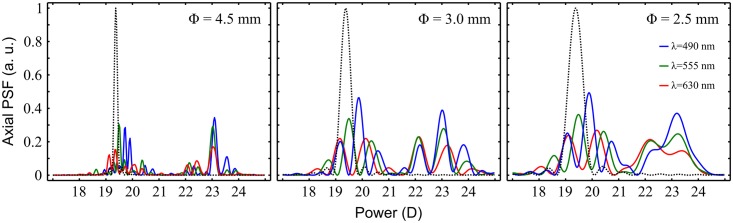
Theoretical axial PSFs provided by a FIOL. Results for a lens with distance power 19.5 D (*Ad* = +3,5D) with different pupil diameters (Φ) and three wavelengths: λ = 490 nm (blue line); λ = 555 nm (green line), and λ = 630 nm (red line). In each plot, the dotted lines are the PSFs (λ = 555 nm) of a monofocal 19.5 D IOL.

The numerical axial PSFs provided by the designed FIOL for different wavelengths (λ) and three different pupil diameters (Φ) are shown in Fig 3 in comparison with the irradiances of a monofocal IOL with the same dioptric power 19.5 D and the same shape factor. As can be seen, for each wavelength the FIOL produces two main foci surrounded by numerous secondary foci that partially overlap each other for different wavelengths. The result is that both, the near and far foci, have an EDOF under polychromatic illumination.

**Fig 4:**
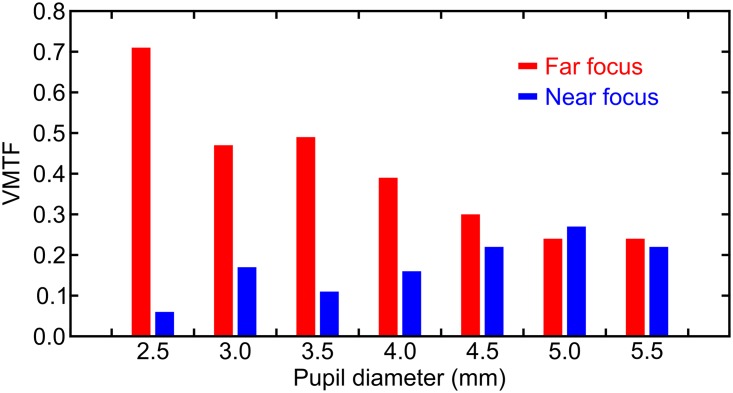
Theoretical visual MTF for the different pupil sizes. These results were computed from the Fourier transform of the monochromatic PSF (the MTF) for the design wavelength λ_0_ = 555 nm, weighted by the neural contrast sensitivity function [21].

Additionally, another objective metric, highly correlated with the visual acuity: the theoretical visual Strehl ratio computed in frequency domain (MTF method) [21], or simply: the Visual MTF (VMTF), was computed for the two main foci (far and near), with different pupil sizes (see Fig 4). As can be seen, despite of being pupil-dependent, the FIOL enhanced the far vision, especially with small pupil sizes.

## Experimental results

**Fig 5:**
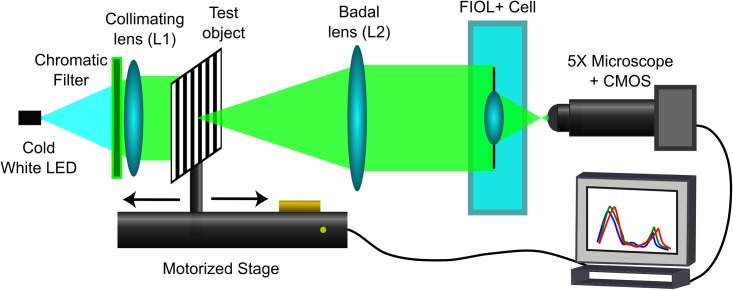
Optical bench for in-vitro testing. The object test was mounted on a linear translation stage. As the FIOL to be tested was placed at the image focal plane of L2 we called it: Badal lens. This configuration guaranteed that the angle subtended by the test object, and consequently the spatial frequency assessed in the TF-MTF, was constant for all vergences and equal to 14 cpd. The retinal image was recorded with an X5 microscope and a CMOS camera.

The optical performance of the FIOL was experimentally tested in vitro with a custom made image forming system that allows the measurement of the polychromatic TF-MTF. A schematic illustration of the experimental system is shown in Fig 5. This setup is similar to one presented previously [22] containing an ISO eye model [23], except for the artificial cornea which has been removed to obtain a better through the focus resolution. The illumination system consists of a white LED (LuxeonTM, V Portable, Alberta, Canada). A band-pass filter was placed behind it to assess the FIOL performance with different wavelengths. The beam was collimated by the lens L1 (focal length: 50 mm). The test object, a grating target of frequency ν = 5 lp mm^-1^, was mounted on a stepping motorized translation stage (travel range 300 mm, accuracy: ±5 μm). The Badal lens L2 was an achromatic lens of focal length: 160 mm. The FIOL prototype was placed in different holders with different pupil sizes and immersed in a wet cell with saline solution. An 8-bit CMOS camera (EO-5012C; Edmund Optics, Illinois, USA); attached to an X5 microscope (focused on the far focal plane of the FIOL) was used to capture the image of object for different vergences. The spatial frequency of the grating corresponds to an object of size 20/40 (0.3 logMAR) in a visual acuity (VA) chart, and is constant at all object vergences.

The object plane was displaced along the optical axis to generate vergences, ranging from -1D to +6D in steps of 0.04D. Vergences were measured from the object focal plane of L2, being positive for displacements towards L2, and negative for displacements in the opposite direction. For each position of the object, the retinal image was stored and analyzed in a totally automatic procedure. The movements of the translation stage and the processing of the retinal images were controlled by custom software programmed in LabView. The MTF for each object vergence was obtained from the calculation of the loss of contrast of the image of the test object. A detailed description of the setup performance can be found elsewhere [22].

**Fig 6:**
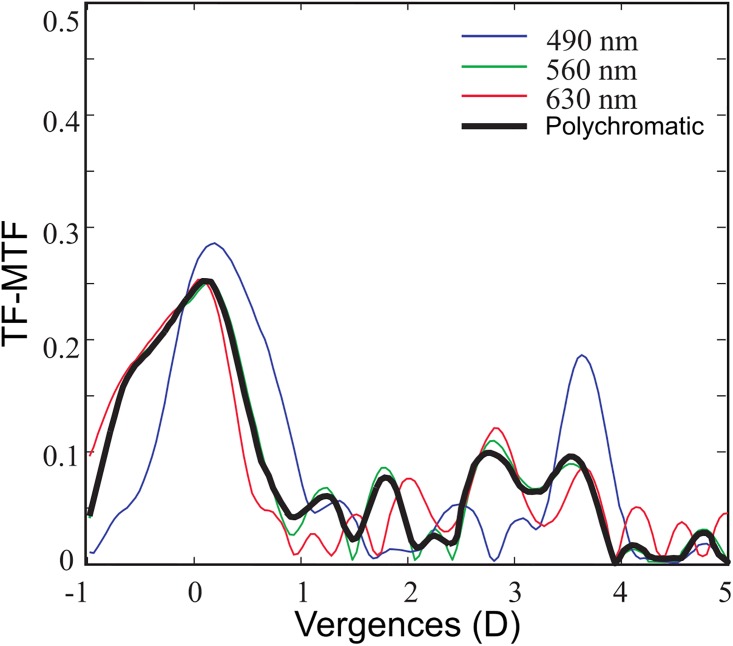
Experimental TF-MTF. FIOL’s TF-MTF for 14 cpd obtained in the optical bench (Fig 6) with 4.5 mm pupil for different wavelengths. Zero defocus corresponds to far vision.

Fig 6 shows the experimental TF-MTF, measured for an aperture of 4.5 mm and three different wavelengths: 490 nm, 560 nm, and 630 nm.

As predicted by the theoretical axial PSF (Fig 3) the TF-MTF curve presents several peaks for the different wavelengths distributed along the whole range of vision. Finally, for evaluating the polychromatic imaging in the eye, these monochromatic (RGB) MTFs were combined numerically, weighted by the spectral sensitivity function of the human eye under daylight conditions V (λ) [24], the spectral content of the illumination source, and the FIOL material transmission. The result, represented by the black line represents in Fig 6., is a compound focal volume with EDOF.

## Discussion and conclusions

In the present study, a new hybrid diffractive/refractive multifocal IOL was presented and evaluated in-vitro. The theoretical design of this lens is based on a general method [10] that includes diffractive profiles having different aperiodic distributions of annular zones. Here, a proof of concept was developed using the triadic Cantor set fractal distribution. In our design, we have found that the Cantor function with *S* = 2 and with *K* = 3, provides the simplest multifocal structure for a FIOL, in which the refractive and diffractive properties of the lens are optimized. In fact, as shown in Ref. [11], as *S* becomes larger in a FZP, an increasing number of subsidiary (diffractive) foci are generated, which means that, in designs with *S* = 3, the diffractive effects would become more predominant over the refractive ones, which would result in a loss of light efficiency compared to designs with *S* = 2.

The proposed lens (FIOL) is a center-distance EDOF design that provides a clear dominance of the far focus with different pupil sizes. In fact, the theoretical results presented in Fig 4, show that despite of some degree of pupil-dependence, the highest value of MTF was achieved for the far focus for almost all considered pupil diameters. Opposite results, i.e.; lenses with a clear dominance of the near focus, were obtained in a preliminary study [25] (in Spanish). In that work we investigated the performance of a design in which the zones were interchanged with respect to those shown in Fig 1.

The polychromatic behavior of the lens was assessed both theoretically, and experimentally in a dedicated optical setup. We have shown that, thanks to its hybrid nature, the FIOL has two principal foci, intended to provide far and near vision, and a series of secondary foci around them, that give an EDOF to each main focus, improving intermediate vision. Moreover, thanks to these secondary foci, the FIOL has a reduced chromatic aberration because under polychromatic illumination there is a partial overlapping between them for the different wavelengths (see Fig 3). In the analysis of the experimental results reported in Fig 6. It should be taken into account that both, the cutoff frequency, and the values of the MTF obtained in the test bench for the FIOL without cornea are lower than the cutoff frequency provided by an artificial eye with the cornea lens and the FIOL [25]. Futhermore, because of the hybrid nature of the far and near foci, and based on the results recently reported by Nakajima et al. [26] for monofocal refractive IOLs, it can be expected that the visual performance of eyes implanted with FIOLs will be similar to that of phakic eyes when some extent of higher-order aberration exists. At this point, it is important to note that this behavior is different from other diffractive multifocal IOLs, which have elevated levels of chromatic aberration of opposite sign [8, 27].

We want to emphasize that the design parameters of the FIOL allow customization. In fact, a FIOL can be designed to match the patient’s *Ad*, and visual needs; for instance: ratio between the near and far intensities can be modified by the lacunarity in the Cantor set (see Fig. 3 Ref. [19]). This parameter also allows controlling the number of foci of the FIOL. Therefore, the flexibility in the FIOL design can be considered an advantage over other multifocal IOL models. Moreover, other fractal profiles can be used to address other particular needs.

Some limitations of this study will be addressed in the future. Further studies should involve other in vitro optical quality measurements of the FIOL, such as: the use of other (foldable) materials for the lens construction, and the effect on the merit functions (PSF, MTF, and TF-MTF) of the FIOL decentration and tilt [28]. Moreover, improvements in the reported results could be expected with different aspheric designs on the base lens intended to the correction of spherical aberration [29]. Finally, a clinical evaluation of patients who have had implantation of the FIOL is required to determine the visual impact of our design on the patient’s quality of vision, particularly, to assess how the multifocal action of the lens affects the contrast sensitivity at several distances.

## Supporting information

S1 Dataset Axial PSFs data, computed at 0.03D intervals as represented in Fig 3. (XLSX)
